# Elevated Oxidative Stress in Patients with Coexisting Multiple Sclerosis and Migraine: A Cross-Sectional Study

**DOI:** 10.3390/antiox14050511

**Published:** 2025-04-24

**Authors:** Iwona Rościszewska-Żukowska, Marek Biesiadecki, Mateusz Mołoń, Aleksandra Rożek, Halina Bartosik-Psujek, Sabina Galiniak

**Affiliations:** 1 Faculty of Medicine, University of Rzeszów, Warzywna 1a, 35-310 Rzeszów, Poland; mbiesiadecki@ur.edu.pl (M.B.); hbartosik@ur.edu.pl (H.B.-P.); 2 Faculty of Biology and Nature Protection, University of Rzeszów, Zelwerowicza 4, 35-601 Rzeszów, Poland; mmolon@ur.edu.pl (M.M.); ar113963@stud.ur.edu.pl (A.R.)

**Keywords:** migraine, multiple sclerosis, lipid peroxidation

## Abstract

One potential association that is gaining increasing attention is the link between multiple sclerosis (MS) and migraine, which are suggested to frequently coexist in young patients. This is the first study to analyze the levels of multiple markers of oxidative stress in sociodemographically similar groups of patients with migraine, MS, and both MS and migraine. A single cross-sectional study was conducted at the Department of Neurology, Rzeszów University. The study included 110 participants, comprising 26 healthy controls, 24 subjects with migraines, 30 with MS, and 30 with both MS and migraine. Oxidative stress markers were measured in patients’ serum. Patients with MS and migraines had statistically elevated levels of 3-nitrotyrosine, Amadori products, 4-hydroxy-nonenal, and oxidative damage to amino acids. Moreover, we observed reduced levels of thiol groups and total antioxidant capacity in the serum of patients with MS and migraines compared to healthy controls. The co-occurrence of migraines in MS leads to greater oxidative stress than MS alone. The impact of chronic oxidative stress on both MS and migraines may exacerbate symptoms and deteriorate the quality of life.

## 1. Introduction

Multiple sclerosis (MS) is a chronic autoimmune disease characterized by neurodegeneration and demyelination of the central nervous system, primarily affecting young adults and exhibiting a variable disease course. Despite significant progress in understanding this disease, many aspects of its onset and development remain unclear [1]. In recent years, researchers’ interest has focused on potential connections between MS and other neurological disorders as well as environmental factors [2]. In MS, the gut microbiome is considered a possible mediator of environmental risk factors, and research indicates that disease-modifying therapies (DMTs) may impact the makeup of the gut microbial community [3]. Moreover, epigenetic mechanisms are believed to contribute to the development of MS, with environmental factors driving cell-type-specific alterations in gene expression [4,5].

One of the emerging associations receiving growing interest is the link between MS and migraine, which are believed to frequently coexist in many young patients [6,7,8]. Although the two conditions differ in symptoms and pathophysiology, there are suggested similarities and common mechanisms that may indicate a connection between them [9]. Migraine is one of the most common neurological disorders, whereas MS is the leading non-traumatic cause of neurological disability in young adults, affecting more than 2.2 million people worldwide [10,11].

Furthermore, there is growing interest in the role of oxidative stress in the pathogenesis of MS [12,13]. Oxidative stress, caused by an imbalance between reactive oxygen species (ROS) and antioxidant defenses, has been linked to various neurological disorders, including MS [14]. In migraines, disturbances occur in the balance between pro-oxidative and antioxidative mechanisms, leading to an increased presence of ROS and tissue damage. Studies have shown that patients with migraines often exhibit elevated levels of oxidative stress biomarkers, such as lipid peroxidation, free radical concentration, and altered antioxidant enzyme activity [15,16]. Additionally, oxidative stress can result in mitochondrial dysfunction, which is particularly relevant as mitochondria play a crucial role in energy production processes in the brain [17]. Moreover, there is evidence suggesting that migraine triggers, such as emotional stress, hormonal changes, or diet, may increase oxidative stress through various mechanisms, including increased activity of NADPH oxidase enzymes, which are the main source of ROS in the brain [18,19].

In addition to their immunomodulatory roles, DMTs used in MS patients have been shown to influence oxidative stress levels, with some agents exerting antioxidant effects (e.g., interferon-beta, glatiramer acetate), while others may have variable or even pro-oxidant actions depending on dosage and patient profile [20,21]. This aspect is particularly important in studies analyzing redox profiles in MS patients, as treatment-related effects may confound biomarker interpretation.

Moreover, although MS and migraine differ in clinical course and primary pathomechanisms, both conditions involve oxidative and nitrosative stress, neuroinflammation, and mitochondrial dysfunction [22,23]. Despite frequent comorbidity, the pathophysiological links between MS and migraine remain unclear. Our study addresses this gap by comparing oxidative stress markers in well-matched groups of patients with MS, migraine, and their coexistence in order to better understand possible additive or synergistic redox-related mechanisms.

The precise mechanisms underlying the relationship between oxidative stress and migraines remain the subject of intense research. Further understanding of these relationships may lead to the development of more effective anti-migraine therapies that also target oxidative stress. Moreover, understanding the role of oxidative stress in the context of MS and its potential associations with migraines may have significant implications for DMTs in MS and the prevention of both diseases. To our knowledge, this is the first study to comprehensively assess multiple oxidative stress markers in sociodemographically similar groups of patients with migraine, MS, and both MS and migraine.

## 2. Materials and Methods

### 2.1. Ethical Considerations and Protocol Approval

This investigation adhered strictly to ethical guidelines, receiving approval from the University of Rzeszów’s Bioethics Committee in Poland (Approval No. 2/02/21). All methodologies involving human subjects complied with both the institutional and national ethical standards, as well as the principles outlined in the 1964 Declaration of Helsinki and its later modifications. Informed consent was properly acquired from all participants.

### 2.2. Participants

A total of 60 patients with relapsing-remitting multiple sclerosis (RRMS), all treated with DMTs matched with DMTs type at the MS Centre of the Clinical Hospital No. 2 in Rzeszow, Poland, were included in the study: 30 patients with RRMS and 30 patients with both RRMS and migraine. To the study group we also included 24 episodic migraine patients and 26 healthy controls. Recruitment took place from 1 January 2023 to 1 July 2023. All participants were diagnosed and assessed at the Neurology Outpatient Clinic. RRMS was diagnosed according to McDonald’s criteria 2017, migraine was diagnosed by headache specialist according to the 2018 ICHD-3 headache classification criteria [24,25]. The following exclusion criteria were established: less than 18 years, more than 60 years, other than RRMS type: primary progressive multiple sclerosis (PPMS) and secondary progressive multiple sclerosis (SPMS), change of DMTs, relapse and steroid treatment, prophylactic migraine treatment, and antioxidant supplementation during the last 120 days: vitamin C and E, co-enzyme Q10, selenium, melatonin, carotenoids, flavonoids, and lipoic acid. Participants were excluded if they had chronic migraine, other primary headaches, secondary headaches, cranial and cervical vascular disorders, epilepsy, mental disorders, chronic sinusitis, or hypertension.

In accordance with the study protocol, vital signs and biometric parameters (such as weight and body mass index (BMI)) were assessed, and both physical and neurological examinations were conducted. In RRMS study groups, the Expanded Disability Status Scale (EDSS) was administered by a trained neurologist. Additionally, the following tests were conducted: the Nine-Hole Peg Test (9HPT)—a standardized, quantitative measure of finger dexterity; the Timed 25-Foot Walk (T25FW)—a quantitative test of mobility and leg function; and the written Symbol Digit Modalities Test (SDMT)—a test used to detect cognitive impairment. All participants were examined in the questionnaire with demographic and clinical migraine and MS data.

### 2.3. Reagents and Methods

Analytical-grade reagents for this study were sourced exclusively from Sigma-Aldrich, Poznań, Poland. Absorbance measurements were performed using a Tecan Infinite 200 PRO multimode reader (Tecan Group Ltd., Männedorf, Switzerland), with each measurement replicated three times, standardizing results to 1 mg of total protein, unless specified otherwise.

#### 2.3.1. Blood and Sampling

Blood samples were collected from fasting subjects between 8 and 10 AM using the Sarstedt S-Monovette system, followed by centrifugation at 1000× *g* for 10 min at 4 °C. Then, the serum was stored at −80 °C, with a maximum storage period of three months, and thawed only once immediately prior to analysis.

#### 2.3.2. Protein Assay

Estimated using the Lowry method, involving the application of Lowry reagent to a 96-well plate, followed by serum, and incubation with the Folin–Ciocalteu reagent. Absorbance was measured at 750 nm [26].

#### 2.3.3. Thiol Groups

Evaluated using the Ellman method, with absorbance measured after 1 h of incubation at 37 °C at 412 nm in a reaction involving serum and 5,5′-dithiobis-(2-nitrobenzoic acid) in a phosphate buffer [27]. The thiol group concentration was calculated based on a standard curve, with glutathione used as the standard, and expressed as nmol/mg protein.

#### 2.3.4. Amadori Products

Quantified by the Johnson method using nitro blue tetrazolium, with absorbance measured at 525 nm after 2 h of incubation at 37 °C [28]. The Amadori products were quantified using an extinction coefficient of 12,640 M^−1^ cm^−1^ for monoformazan [29]. Measurements were made in duplicate.

#### 2.3.5. 3-Nitrotyrosine

Assessed using an enzyme-linked immunosorbent assay (ELISA) kit following the manufacturer’s guidelines (Immundiagnostik AG, Bensheim, Germany).

#### 2.3.6. Total Antioxidant Capacity (TAC)

TAC was assessed using two methods. Formed 2,2′-azino-bis(3-ethylbenzothiazoline-6-sulfonic acid) radical (ABTS^•^) evaluated for its scavenging capacity in serum samples, with results expressed in Trolox equivalents (μmol TE/L) [30].

The ferric reducing antioxidant power (FRAP) was measured colorimetrically, with results also expressed in Trolox equivalents (μmol TE/L) [31].

#### 2.3.7. Lipid Peroxidation

Two markers of lipid peroxidation were measured—malondialdehyde (MDA) and 4-hydroxy-nonenal (4-HNE). For MDA determination, serum samples were mixed with 200 μL of a mixture containing 0.37% thiobarbituric acid and 15% trichloroacetic acid in 0.25 M HCl to precipitate the protein. The samples were incubated at 100 °C for 40 min. After centrifugation, the absorption of the supernatants was measured at a wavelength of 532 nm. The MDA concentration in blood serum was expressed in μmol/L. The results were calculated using an absorption coefficient for MDA of 1.56 × 10^5^ M^−1^cm^−1^ [32].

Moreover, 4-HNE was assayed using an ELISA kit following the manufacturer’s guidelines (Wuhan Fine Biotech Co., Ltd., Wuhan, China).

#### 2.3.8. Advanced Glycation End Products (AGEs)

The estimation was performed using a spectrofluorimetric method to measure novel glucose-derived fluorescence in serum [33,34], with excitation and emission wavelengths set at 350 nm and 440 nm, respectively.

#### 2.3.9. Content of Tryptophan, Dityrosine, Kynurenine and N’-Formylkynurenine

The concentrations of serum tryptophan, dityrosine, kynurenine, and N’-formylkynurenine were estimated based on their fluorescence at the wavelengths of 295/340 nm, 330/415 nm, 365/480 nm, and 325/434 nm, respectively [35].

### 2.4. Statistical Analysis

Data are presented as the mean ± standard deviation (SD), and statistical results were supplemented with 95% confidence intervals (95% CI) where applicable. The normality of the distributions was tested with the Shapiro–Wilk test. To determine statistical significance, the Kruskal–Wallis or Mann–Whitney U test was applied, and the analysis was carried out using the STATISTICA software (version 13.3, StatSoft Inc., Tulsa, OK, USA) for all analyses.

## 3. Results

Our study involved 110 participants, including 26 healthy individuals, 24 with diagnosed episodic migraine, 30 with MS, and 30 patients with both MS and confirmed episodic migraine. The basic clinical data of the individuals recruited for the study are presented in Table 1. No differences were observed in the age and BMI of the study participants. Similarly, there were no differences observed in MS duration, types of DMTs, DMT duration, or clinical test results between the MS and MS with migraine groups.

**Figure 1:**
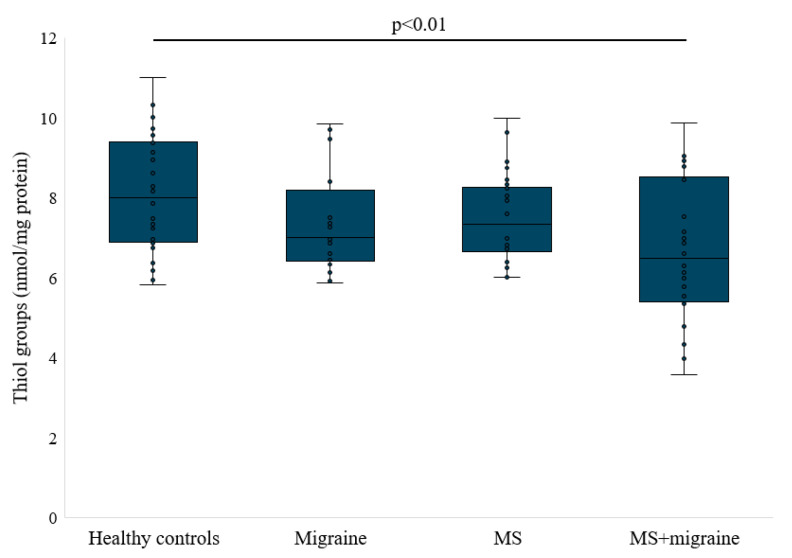
Thiol groups concentration in studied groups.

**Figure 2:**
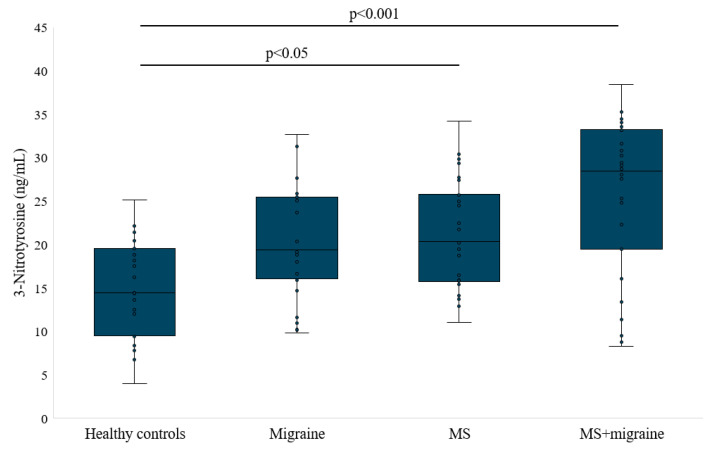
3-nitrotyrosine concentration in studied groups.

**Figure 3:**
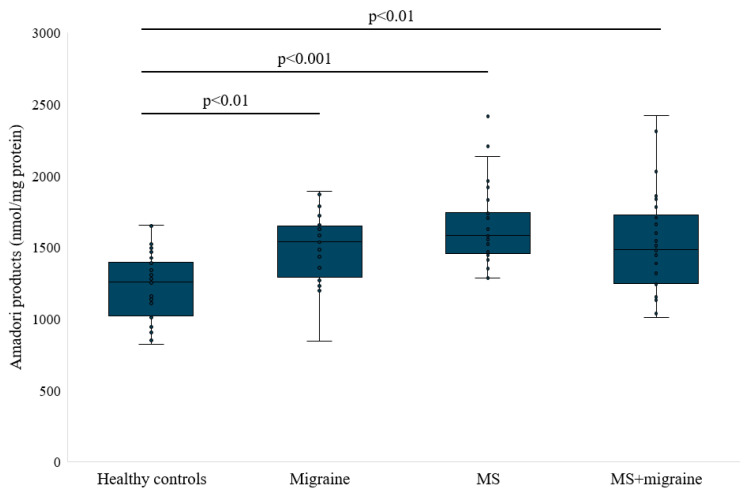
Amadori products concentration in studied groups.

**Figure 4:**
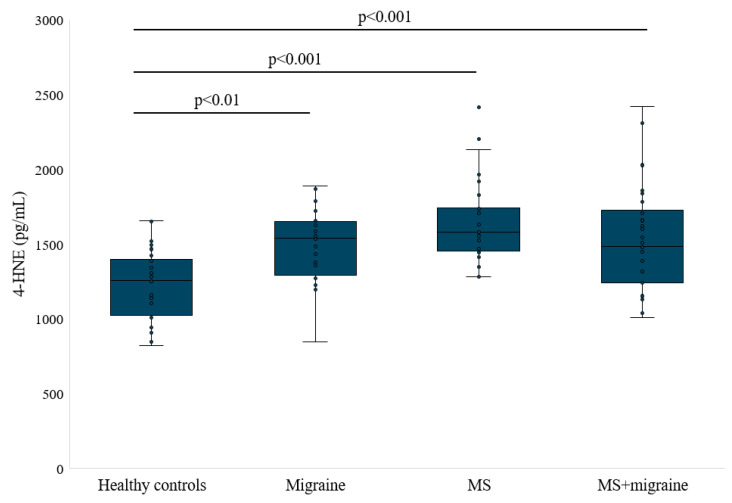
4-HNE concentration in studied groups.

Table 2 and Figure 1, Figure 2, Figure 3 and Figure 4 depict the results of oxidative stress markers. Thiol functional groups are crucial in reducing radicals and other toxic electrophiles. The statistical significance of the Kruskal–Wallis test for the overall comparison was *p* = 0.0118. Post hoc analysis revealed that the content of thiol groups was significantly lower in participants with MS and migraine than in the group of healthy individuals (*p* = 0.005). No significant difference in thiol group concentration was noted between the other groups studied.

3-nitrotyrosine in proteins is generated through the rapid reaction between tyrosyl radicals and ^•^NO_2_ and is used to test the oxidative and nitrosative stress of the body. The Kruskal–Wallis test indicated a highly significant difference across the groups, with a *p*-value of <0.001. Post hoc analysis revealed that the concentration of 3-nitrotyrosine was increased in patients with MS and MS with migraine compared to healthy individuals (20.98 ± 6.22 [95% CI: 18.65–23.29] and 25.99 ± 8.77 [95% CI: 22.71–29.26] vs. 14.8 ± 5.59 [95% CI: 12.54–17.05] ng/mL, *p* = 0.026 and *p* < 0.001, respectively, Figure 2). When comparing patients with both MS and migraine to those with migraine alone (20.46 ± 6.39 [95% CI: 17.76–23.16], we observed higher levels of several oxidative stress markers in the comorbid group. Notably, the concentration of 3-nitrotyrosine was greater, but non-significantly in the MS + migraine group than in the migraine-only group.

Amadori products can be measured to assess the extent of glycation in the body. Because glycation occurs naturally in the body but can also be increased in pathological conditions such as neurodegenerative diseases, measuring Amadori products can be useful as a biomarker for these conditions. Additionally, Amadori products may contribute to the pathogenesis of these diseases by generating ROS and promoting oxidative stress, leading to protein and tissue dysfunction [36]. In Figure 3, we present the concentration of these products in the serum of study participants. In study participants with migraine, MS, and MS and migraine, the concentration of Amadori products was statistically higher than in healthy participants (1488.89 ± 249.9 [95%CI: 1383.4–1594.4], 1641 ± 269.65 [95%CI: 1540.3–1741.7], and 1512.51 ± 357.04 [95%CI: 1379.2–1645.8] vs. 1224.77 ± 233.86 [95%CI: 1130.3–1320] nmol/mg protein, *p* < 0.001).

Markers of lipid peroxidation are substances used to assess the process of lipid peroxidation in the body. No difference in MDA concentration was found among the study groups (Table 2). Statistical analysis showed a significant difference in the concentration of 4-HNE across the entire study group (*p* < 0.001). 4-HNE concentration was statistically increased in the serum of participants with migraine, MS, and MS with migraine compared to controls (404.7 ± 89.54 [95% CI: 366.9–442.5], 455.72 ± 77.42 [95% CI: 426.8–484.6], and 456.95 ± 141.6 [95% CI: 402.2–511.9] vs. 311.45 ± 52.46 [95% CI: 290.3–332.6] pg/mL).

The TAC reflects the body’s ability to counteract oxidative stress by maintaining a balance between ROS and antioxidants. A higher TAC level indicates a greater capacity to neutralize the harmful effects of oxidative stress. While there was no difference in TAC levels determined by FRAP, analysis using ABTS^•^ showed that TAC was reduced in patients with MS and MS with migraine compared to healthy participants. Furthermore, TAC in the MS and migraine group was lower than in participants with migraine alone (Table 2). These findings suggest an additive or synergistic oxidative burden in patients with both conditions. Fluorescence measurements are commonly used to determine AGEs and other compounds such as dityrosine, N’-formylkynurenine, kynurenine, and tryptophan. Levels of AGEs, tyrosine, N’-formylkynurenine, and kynurenine were higher in patients with migraine, MS, and MS with migraine than in healthy individuals. Conversely, tryptophan levels were lower in the MS with migraine group than in healthy controls (Table 2).

## 4. Discussion

The coexistence of migraine and MS has been a subject of discussion, with uncertainty remaining about whether migraine is merely a comorbid condition or if there is a causal link between the two diseases. Additionally, the role of oxidative stress in both migraine and MS has been explored in numerous studies, but the findings appear to be inconclusive. In our study in episodic migraine patients, the concentration of thiol groups was not different than healthy controls. Consistent with our findings, no significant difference was observed in the levels of total thiol, native thiol, and disulfide in children diagnosed with migraine in a study by Kurt et al. [37]. On the other hand, the concentration of thiol was significantly reduced in migraine patients than in controls in a study by Eren et al. [38]. In our assessment, interpreting this effect unambiguously is challenging, but it could be influenced by genetic factors. The studies we reviewed were conducted in Europe, while others were from Asia. Therefore, comprehensive genetic and epigenetic research will undoubtedly be necessary in the future to definitively determine even this effect.

Although significantly increased nitrate and decreased nitrite levels were observed in migraineurs during the headache-free period [39], surprisingly, we found no difference in the concentration of 3-nitrotyrosine in the serum of migraine patients compared to healthy controls. However, it is important to note that there was a long interval since the last migraine attack in our study group. Increased biomarkers of nitrosative in platelets may be important in migraine patients, especially during attacks. The increase of nitric oxide metabolites in platelets during attacks supports the opinion that nitric oxide may play a modulatory role in biological processes, particularly by vasodilatation in migraine attacks [40]. It is suggested that migraine may be associated with the process of glycation, which is in line with our results of higher concentrations of AGEs and Amadori products and can lead to damage to blood vessels and nerves, which in turn may contribute to the occurrence of migraines or exacerbate their severity [41]. However, the relationship between glycation and migraine has not yet been fully understood and requires further research.

Lipid peroxidation in migraineurs occurs secondary to the oxidative and nitrative stress. Serum MDA levels of patients with migraine were significantly elevated among subjects with chronic migraine compared to episodic migraineurs and control subjects [42,43]. We found no differences in the MDA concentration, but the level of 4-HNE was elevated in episodic migraine patients.

Our results of any differences in TAC level between migraine and healthy controls are in accordance with others. Similarly, studies have shown no significant differences in total oxidant status, total antioxidant status, or oxidative stress index values in patients with migraine [15,38]. However, chronic migraineurs had lower mean TAC values than episodic migraineur patients and controls in a study by Togha et al. [43]. Likewise, lower TAC measured by the FRAP method was noted in chronic migraineurs than in healthy controls [44]. We noticed increased concentrations of dityrosine, N-formylkynurenine, and kynurenine in the serum of migraineurs, which indicates post-translational modifications of proteins. The precise role of post-translational protein modifications in migraine remains unclear and requires further investigation. The elevated oxidative stress in migraine mainly results from the activation of the immune-neurologic system, neuroinflammation, changes in mitochondrial activity, antioxidant deficiency, and the role of oxidative mediators in pain mechanisms [43]. These processes can mutually potentiate, contributing to the exacerbation of migraine symptoms.

In migraine, activation of the immune-neurological system occurs, resulting in the release of pro-inflammatory cytokines such as tumor necrosis factor-alpha and interleukins, especially interleukin-1 beta and interleukin-6. These cytokines can stimulate the production of ROS in nerve cells and endothelial cells [45]. Neuroinflammation, characterized by increased activation of microglia, can also contribute to increased oxidative stress by releasing ROS. Changes in mitochondrial function have been observed in migraine, which may lead to increased ROS production due to mitochondrial damage [46]. Moreover, oxidative stress-related DNA damage was found to be significantly higher in migraine patients than in the control group [15]. High levels of ROS can directly participate in pain mechanisms by activating pain receptors such as the transient receptor potential vanilloid 1, transient receptor potential ankyrin 1, and purinergic receptors, all of which are known to play critical roles in nociceptive signaling, and inducing inflammation in the brain and blood vessels [47]. MS may be affected by environmental and genetic factors, and oxidative stress may be an important factor associated with the development of demyelination. In the present study, the level of thiol groups was similar in MS patients and healthy controls, but all MS patients were clinically stable and free of relapses at least during the last 3 months. Literature data indicate that MS is characterized by reduced thiol group levels [48,49,50,51]. Moreover, serum total thiol and native thiol levels were significantly lower in relapsed patients compared to those in remission [52]. Furthermore, no difference was observed in carbonyl proteins, another marker of protein oxidation, between MS patients and healthy controls [53].

In our study, we noted an increased concentration of 3-nitrotyrosine in the serum of MS compared to healthy subjects. Similar results were reported previously [51]. Moreover, nitrates and nitrites were higher in MS subjects than in healthy participants in a study by Obradovic et al. [54]. Similarly, the levels of nitrite/nitrate in the cerebrospinal fluid of MS patients tend to be higher compared to those in healthy individuals [55].

The levels of Amadori products and AGEs were elevated in MS patients compared to healthy individuals. Similarly, serum glycation markers such as carboxymethyllysine and carboxyethyllysine in patients with MS were increased compared to control participants in a study by Damasiewicz-Bodzek et al. [56]. In MS patients, increased plasma pentosidine and carboxymethyllysine levels were identified using immunohistochemical techniques in postmortem hippocampal samples [57,58]. Elevated oxidative stress in patients appears to contribute to the progression of glycoxidative protein damage, and the measurement of glycation end products may serve as a marker for evaluating the clinical condition of MS patients [59]. Methylglyoxal-derived AGEs accumulating on proteins of the myelin sheath may contribute to altered antigen presentation, resembling exogenous antigens and initiating an autoimmune response [60]. In the present study, 4-HNE concentration was significantly increased, while MDA concentration was similar in MS patients and control subjects. Literature data indicate that MDA levels were significantly elevated in MS patients, particularly during relapses [51,54,61]. The levels of 4-HNE were elevated in the cerebrospinal fluid of RRMS, PPMS, and SPMS patients in the study by Podbielska et al. [62]. 4-HNE is highly diffusible and exhibits strong toxicity toward central nervous system cells, including oligodendrocyte precursor cells and cerebral endothelial cells. Moreover, the abundance of 4-HNE-positive macrophages surrounding blood vessels suggests that increased perivascular 4-HNE levels may play a role in the dysfunction of the blood–brain barrier [63]. Oxidative stress resulting from lipid peroxidation is associated with inflammation, demyelination, and neurodegeneration in MS [64,65].

We detected decreased serum TAC in MS patients. TAC measured by FRAP was also lower in MS patients compared to healthy controls [49]. Moreover, the total radical-trapping antioxidant parameter was also lowered in MS patients as compared to healthy controls [48,53]. The increased oxidative stress in MS results from a combination of factors, including autoimmune attack on neural tissue, decreased activity of antioxidant enzymes, mitochondrial damage, metabolic disorders, and endoplasmic reticulum stress [66]. These mechanisms mutually reinforce each other, contributing to further damage of nerve cells and leading to disease progression.

An important factor to consider is that all MS patients in our study, including those with coexisting migraine, were under DMTs treatment. Although the types and durations of therapies were comparable between the MS and MS + migraine groups, the observed differences in oxidative stress markers may be partially modulated by DMT-related effects. For example, interferon-beta and glatiramer acetate have demonstrated antioxidant capacities, while newer agents like fingolimod and dimethyl fumarate may act through mitochondrial pathways or modulate ROS production depending on the cellular context [13,67,68]. It is plausible that the cumulative burden of oxidative stress in the MS + migraine group results not only from disease coexistence but also from complex interactions with treatment-related metabolic changes.

A recent study by Hamamcı et al. found that the activity of antioxidant enzymes and total antioxidant status values were lower in RRMS patients with migraine compared to those without migraine. In their study, the total oxidant status and oxidative stress index values of the RRMS patients with migraine were higher than those without migraine [69]. Therefore, it appears that the co-occurrence of migraine in MS leads to greater oxidative stress than in MS alone. Notably, comparison between the MS + migraine and MS-only groups revealed distinct redox profiles, including further reduced TAC and slightly elevated 3-nitrotyrosine levels. These alterations, along with differences from the migraine-only group, suggest additive oxidative effects and point toward overlapping mechanisms such as mitochondrial dysfunction, neuroinflammation, and disrupted antioxidant defenses. This supports the view that the co-occurrence of MS and migraine is not incidental but may influence disease pathophysiology, symptom burden, and patient outcomes. Strategies to reduce oxidative stress, such as an antioxidant-rich diet and supplementation, may be helpful for both MS and migraine patients. Also, ketone bodies derived from the ketogenic diet may improve mitochondrial metabolism, reduce neuronal excitability, decrease neuroinflammation, and produce mitochondrial-resistant oxygen species [70]. This study underscores the complex interplay between oxidative stress, glycation, and antioxidant responses in neurological and inflammatory conditions, highlighting potential biomarkers like 3-nitrotyrosine, 4-HNE, and TAC for further investigation in these disease contexts.

This study has several notable strengths. First, it is the first to comprehensively analyze multiple oxidative stress markers in sociodemographically similar groups of patients with MS, migraine, and both conditions, allowing for more precise comparisons. Second, the study design includes a healthy control group, providing a clear reference for assessing oxidative stress alterations. Third, the use of multiple oxidative stress biomarkers, including 3-nitrotyrosine, Amadori products, 4-HNE, and oxidative damage to amino acids, provides a comprehensive evaluation of oxidative imbalance. Additionally, the findings contribute to the growing body of evidence on the role of oxidative stress in MS and migraine, emphasizing the potential impact of chronic oxidative stress on disease progression and quality of life. Nevertheless, several limitations should be mentioned. First, its cross-sectional design prevents the establishment of causal relationships between oxidative stress markers and the co-occurrence of MS and migraines. Longitudinal studies are needed to assess the temporal dynamics of oxidative stress in these conditions. Second, the relatively small sample size may limit the generalizability of the findings, and larger cohorts are required to confirm these results. Another limitation of the study is the predominance of women in the study groups—this sex imbalance may limit the generalizability of the findings, particularly to male patients. Another limitation of our study is the lack of direct analysis of the redox-modulating effects of specific DMTs. Although we ensured that treatment types and durations were comparable between MS and MS + migraine groups, individual variations in drug metabolism and pharmacodynamics could have influenced oxidative stress markers. This potential confounder should be addressed in future studies that stratify patients by specific treatment and include pre-treatment biomarker baselines. Finally, oxidative stress markers were measured in serum, which may not fully reflect oxidative processes occurring in the central nervous system. Future research should incorporate cerebrospinal fluid analysis or advanced neuroimaging techniques to better understand the role of oxidative stress in MS and migraine pathophysiology. In addition to these limitations, it is also important to consider potential selection bias related to patient recruitment from a single clinical center, which may affect the external validity of the results. Although validated clinical and laboratory tools were used, inter-individual variability and unmeasured confounding factors—such as diet, stress levels, or undetected low-grade inflammation—may have influenced oxidative stress parameters. Future studies should consider a multicenter design and broader patient stratification (e.g., by disease stage, comorbidities, or treatment duration) to improve the robustness and generalizability of the findings. Moreover, the observed changes in oxidative stress markers may offer potential for clinical application, such as the development of biomarkers for disease monitoring or personalized therapeutic strategies. These possibilities warrant confirmation in larger, mechanistically oriented studies with longitudinal follow-up.

## 5. Conclusions

The study investigated various protein oxidation markers, including 3-nitrotyrosine and Amadori products, along with markers of lipid peroxidation, thiol groups, and TAC, among individuals with migraine, MS, and both conditions compared to healthy controls.

The concentration of 3-nitrotyrosine was elevated in patients with MS and MS with migraine compared to healthy people, suggesting enhanced oxidative and nitrosative stress. The study also found lower levels of thiol groups in participants with MS and migraine compared to healthy controls, indicating reduced capacity to counteract radicals and toxic electrophiles. Additionally, the concentration of Amadori products, markers of glycation and oxidative stress, was higher in individuals with migraine, MS, and both conditions compared to healthy participants.

Markers of lipid peroxidation, such as 4-HNE, were elevated in participants with migraine, MS, and MS with migraine compared to controls, indicating increased lipid oxidation and potential tissue damage. Furthermore, TAC levels, reflecting the body’s antioxidant capacity, were reduced in patients with MS and MS with migraine compared to healthy participants, indicating a compromised ability to neutralize oxidative stress. Fluorescence measurements revealed higher levels of AGEs and other compounds in individuals with migraine, MS, and both conditions compared to healthy controls, suggesting increased oxidative stress and potential involvement in disease pathogenesis.

Overall, the study highlights the association between oxidative stress and both migraine and MS, suggesting potential implications for disease management and the development of targeted therapies.

## Figures and Tables

**Table 1:** Characteristics of the study cohort.

	Healthy Controls	Migraine	MS	MS+ Migraine	*p*
n	26	24	30	30	
Sex (F/M)	23/3	23/1	22/8	24/6	
Age (years)	38.19 ± 7.7	39.75 ± 7.61	38.63 ± 9.91	37.46 ± 10.85	0.867
BMI (kg/m^2^)	25.86 ± 3.61	25.32 ± 4.91	25.04 ± 5.75	24.92 ± 4.11	0.824
Disease duration from age of MS diagnosis (years)	-	-	8.96 ± 6.43	8.1 ± 5.97	0.623
DMT type, n (%)					
*Teriflunomide*	-	-	3 (10)	3 (10)	
*Interferon beta 1a*	-	-	1 (3.3)	2 (6.7)	
*Interferon beta 1b*	-	-	8 (26.7)	7 (23.3)	
*Glatirameracetate*	-	-	2 (6.7)	2 (6.7)	
*Fingolimod*	-	-	3 (10)	3 (10)	
*Cladribine*	-	-	2 (6.7)	2 (6.7)	
*Dimethylfumarate*	-	-	8 (26.7)	8 (26.7)	
*Ozanimod*	-	-	3 (10)	3 (10)	
Time DMT (years)			3.13 ± 2.36	5.2 ± 4.1	0.069
EDSS (points)	-	-	1.23 ± 0.73	1.61 ± 0.78	0.082
9HT RD (s)	-	-	21.22 ± 3.06	20.37 ± 3.22	0.362
9HT RND (s)	-	-	21.72 ± 3.26	22.4 ± 4.36	0.718
T25FW (s)	-	-	5.16 ± 1.03	5.07 ± 0.91	0.774
SDMT score	-	-	53.18 ± 19.44	53.2 ± 13.2	0.574

9HPT RD—Nine-Hole Peg Test dominant hand; 9HPT RND—Nine-Hole Peg Test non-dominant hand.

**Table 2:** Markers of oxidative stress in serum in participants of the study groups.

		Healthy Controls	Migraine	MS	MS + Migraine	*p*
MDA (μmol/L)	mean ± SD	3.52 ± 0.27	3.61 ± 0.27	3.65 ± 0.38	3.57 ± 0.33	0.479
	95% CI	3.41–3.63	3.49–3.72	3.51–3.79	3.45–3.69	
TAC (FRAP, μmol TE/L)	mean ± SD	169.58 ± 25.63	164.08 ± 22.78	158.53 ± 20.14	153.46 ± 19.92	0.161
	95% CI	159.22–179.93	154.46–173.7	151.01–166.05	146.02–160.89	
TAC (ABTS^•^, μmol TE/L)	mean ± SD	314.33 ± 24.77	301.72 ± 16.08	290.01 ± 17.66 ^a^**	281.32 ± 25.7 ^a^***^, b^*	<0.001
	95% CI	304.32–324.33	294.93–308.51	283.42–296.61	271.73–290.92	
AGEs (a.u./mg protein)	mean ± SD	4.07 ± 0.73	5.1 ± 1.17 ^a^**	4.89 ± 0.95 ^a^*	5.21 ± 1 ^a^***	<0.001
	95% CI	3.77–4.36	4.6–5.59	4.53–5.24	4.84–5.59	
Dityrosine (a.u./mg protein)	mean ± SD	2.64 ± 0.36	3.52 ± 1.19 ^a^***	3.19 ± 0.61^a^**	3.39 ± 0.64 ^a^***	<0.001
	95% CI	2.5–2.79	3.01–4.02	2.97–3.42	3.15–3.63	
N’-formylkynurenine (a.u./mg protein)	mean ± SD	2.94 ± 0.54	4 ± 1.68 ^a^***	3.65 ± 0.82 ^a^**	3.81 ± 0.75 ^a^***	<0.001
	95% CI	2.72–3.16	3.29–4.71	3.34–3.95	3.53–4.09	
Kynurenine (a.u./mg protein)	mean ± SD	4.8 ± 0.81	6.38 ± 1.69 ^a^**	6.04 ± 1.2 ^a^**	6.12 ± 1.31 ^a^**	<0.001
	95% CI	4.48–5.13	5.67–7.09	5.59–6.49	5.63–6.61	
Tryptophan (a.u./mg protein)	mean ± SD	167.35 ± 16.86	161.51 ± 29.06	159.25 ± 23.18	147.7 ± 25.77 ^a^**	0.013
	95% CI	160.54–174.16	149.24–173.78	150.59–167.91	138.07–157.32	

^a^—compared to the control, ^b^—compared to migraine, *—*p* < 0.05, **—*p* < 0.01, ***—*p* < 0.001.

## Data Availability

Anonymized data will be shared by reasonable request from qualified investigators.

## Abbreviations

ABTS—2,2′-azino-bis(3-ethylbenzothiazoline-6-sulfonic acid); AGEs—advanced glycation end products; BMI—body mass index; DMTs—disease-modifying therapies; EDSS—Expanded Disability Status Scale; ELISA—enzyme-linked immunosorbent assay; FRAP—ferric reducing antioxidant power; 4-HNE—4-hydroxy-nonenal; 9HPT—Nine-Hole Peg Test; MDA—malondialdehyde; MS—multiple sclerosis; PPMS—primary progressive multiple sclerosis; ROS—reactive oxygen species; RRMS—relapsing-remitting multiple sclerosis; SDMT—Symbol Digit Modalities Test; SPMS—secondary progressive multiple sclerosis; T25FW—Timed 25-Foot Walk; TAC—total antioxidant capacity.
